# Percutaneous Image-Guided Ablation of Lung Tumors

**DOI:** 10.3390/jcm10245783

**Published:** 2021-12-10

**Authors:** Sadeer J. Alzubaidi, Harris Liou, Gia Saini, Nicole Segaran, J. Scott Kriegshauser, Sailendra G. Naidu, Indravadan J. Patel, Rahmi Oklu

**Affiliations:** 1 Department of Radiology, Mayo Clinic, Phoenix, AZ 85054, USA; skriegshauser@mayo.edu (J.S.K.); naidu.sailen@mayo.edu (S.G.N.); patel.indravadan@mayo.edu (I.J.P.); oklu.rahmi@mayo.edu (R.O.); 2 Alix School of Medicine, Mayo Clinic, Scottsdale, AZ 85259, USA; Liou.harris@mayo.edu; 3 Division of Vascular and Interventional Radiology, Laboratory for Patient Inspired Engineering, Mayo Clinic, Phoenix, AZ 85054, USA; saini.gia@mayo.edu (G.S.); nsegaran2@gmail.com (N.S.)

**Keywords:** lung ablation, percutaneous ablation, cryoablation

## Abstract

Tumors of the lung, including primary cancer and metastases, are notoriously common and difficult to treat. Although surgical resection of lung lesions is often indicated, many conditions disqualify patients from being surgical candidates. Percutaneous image-guided lung ablation is a relatively new set of techniques that offers a promising treatment option for a variety of lung tumors. Although there have been no clinical trials to definitively compare its efficacy to those of traditional treatments, lung ablation is widely practiced and generally accepted to be safe and effective. Especially encouraging results have recently emerged for cryoablation, one of the newer ablative techniques. This article reviews the indications, techniques, contraindications, and complications of percutaneous image-guided ablation of lung tumors with special attention to cryoablation and its recent developments in protocol optimization.

## 1. Introduction

Despite advances in therapy and prevention, lung cancer is persistently the leading cause of cancer death around the world. In 2021 alone, an estimated 131,880 Americans will die from lung cancer [1]. The lack of effective screening methods and delayed detection contribute to poor overall prognosis and limitation of treatment options. Additionally, metastases to the lungs, including those from primary breast and colorectal carcinomas, cause significant morbidity and mortality [2].

Surgical resection of tumors is the standard treatment for early-stage non-small cell lung cancer (NSCLC). Similarly, surgical resection of metastases to the lungs, or metastasectomy, is a widely practiced procedure believed to confer survival and potentially curative benefit [3]. Unfortunately, many contraindications to surgery exist and thus only approximately one-third of stage I/II NSCLC patients qualify for surgical resection [4]. The traditional alternative therapy is radiation via either stereotactic body radiation therapy (SBRT) or conventional fractionation, but these therapies have serious limitations, including the potential for prolonged exposure to radiation [5,6]. In the past 20 years, percutaneous image-guided lung ablation has emerged as an additional alternative treatment with promising outcome data.

Percutaneous lung ablation was first described in 2000 when Dupuy et al. applied computed tomography (CT)-guided radiofrequency ablation (RFA), which was already used to treat liver malignancies, to induce necrosis of lung tumors [7]. Development of other CT-guided techniques soon followed, including the advent of various thermal ablative techniques (i.e., microwave ablation, laser ablation) and non-thermal techniques such as irreversible electroporation, which are all still in use and under investigation today. Recently, beneficial outcomes from the use of cryoablation have been emerging and it is becoming more popular as a thermal ablative technique.

## 2. Methods

This article will review the indications and techniques of percutaneous image-guided ablation of lung tumors with special attention to cryoablation and its recent developments in protocol optimization. Here, we performed a narrative review of studies on percutaneous image-guided ablation of lung tumors. Thus, the literature search was conducted in a non-systematic method. The databases used in the literature search included PubMed, ScienceDirect, Scopus and Google Scholar. Specific keywords and phrases used during the search included lung ablation, percutaneous ablation, cryoablation, and non-small cell lung cancer. No specific inclusion or exclusion criteria of literature was used in this study. Therefore, studies and articles of varying degrees of quality and bias are referenced in this review. Additionally, examples of specific patient cases at the author’s institution are included to support the use of percutaneous image-guided ablation of lung tumors. Lastly, contraindications as well as complications of percutaneous image-guided ablation will be discussed to provide readers with a comprehensive understanding of percutaneous image-guided ablation of lung tumors.

## 3. Indications

### 3.1. Non-Small Cell Lung Cancer

**Figure 1:**
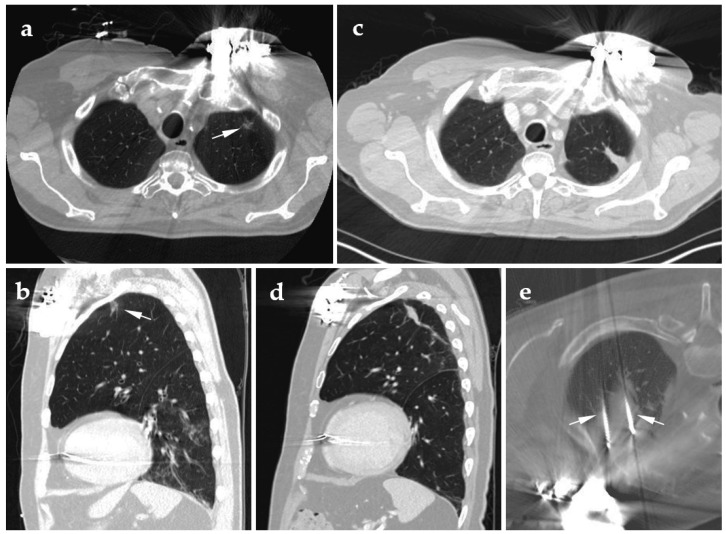
78-year-old male with no significant comorbidities had biopsy-proven lepidic adenocarcinoma consistent with stage 1 NSCLC. As his automatic implantable cardioverter defibrillator would have needed to be removed for SBRT treatment, he was referred to interventional radiology. (**a**) Supine axial pre-ablation CT with the white arrow indicating the lesion to be ablated; (**b**) supine sagittal pre-ablation CT with the white arrow indicating the lesion to be ablated; (**c**) supine axial follow-up CT at 3 years post-ablation; (**d**) supine sagittal follow-up CT at 3 years post-ablation; (**e**) prone axial CT shows the two probes (white arrows) bracketing the lesion during the tumor cryoablation.

**Figure 2:**
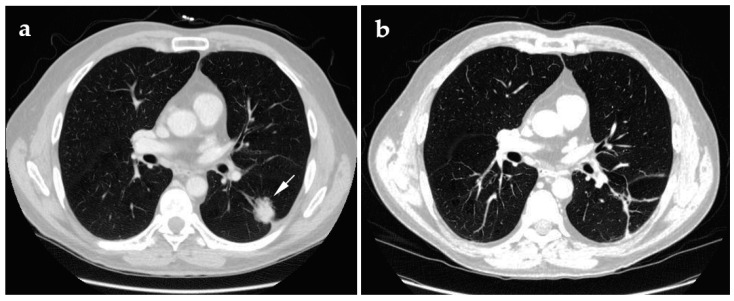
61-year-old male presenting with adenocarcinoma with invasive acinar growth. Endobronchial ultrasound showed no lymph node involvement. (**a**) Supine axial pre-cryoablation CT with the white arrow indicating the lesion to be ablated; (**b**) supine axial follow-up CT at 1-year post-cryoablation.

In addition to SBRT and fractionated radiotherapy, percutaneous ablation is a treatment option for stage I NSCLC patients who are not surgical candidates or refuse surgery (Figure 1 and Figure 2) [8]. Exclusion criteria for surgical resection include cardiorespiratory co-morbidities, insufficient lung function, and age of 75 years or greater [9]. For such patients, curative ablation is generally indicated for tumors measuring 3.5 cm or less in diameter in stage IA or IB NSCLC [10]. As ablation provides only local control, meticulous nodal staging is critical for developing a treatment plan that addresses all lesions. A study found 34% of 2.1–3.0 cm peripheral NSCLC tumors to have lymph node metastases [11]. Imaging alone has limited efficacy for evaluating lymph nodes, leading to frequent under-staging of primary lung cancer patients and potentially improper use of ablation [12].

Table 1 compares 5-year survival and local recurrence outcomes for stage I NSCLC treated with surgical resection, radiofrequency ablation, cryoablation, and other surgical alternatives. Surgical resection and node dissection patients are likely to be upstaged via pathological evaluation, while SBRT and ablation patients are likely to be under-staged due to undetected nodal involvement [13]. This discrepancy at therapy initiation may skew study outcomes to favor resection. Furthermore, while radiation oncology literature considers only the treated area for reporting local recurrence, surgical and radiological literature considers lymph node involvement in the regional area as well. Hence it is imperative to evaluate locoregional recurrence.

**Table 1:** Comparison of 5-year survival, local recurrence rates, and sample ages between resection, traditional surgical alternatives, and lung ablations for stage I NSCLC. No reports of 5-year survival for NSCLC treated by thermal ablation techniques other than those listed have been published.

Modality	5-Year Survival Stage I NSCLC	Local Recurrence	Sample Age (Mean, Median, Range)
Lobar resection [14]	60–80%	3–9%	NA
Sub-lobar resection [15,16]	60–74%	17%	67.3, 68, 20–101 [15] 76.9, NA, 72–85 [16]
SBRT [17,18,19]	42–55%	14%	74.2, NA, NA [18]
External beam radiation [20]	10–27%	50–55%	NA, 70, 34–90
Radiofrequency ablation [21,22]	27–56%	22%	68.5, NA, 17–94 [21] 70, NA, 48–84 [22]
Cryoablation [23]	68%	36% (locoregional)	74.8, NA, 49–85

NA= Not Available.

Of note, the cryoablation study by Moore et al. reported the highest 5-year survival rate of all non-surgical therapies in Table 1 at 68% [23]. Notable also is a study of 160 cryoablation-treated stage I NSCLC patients with an observation period ranging from 12–68 months (median 23 months) that reported only 1 (0.6%) local and 7 (4.3%) total recurrences [24]. A meta-analysis on SBRT-treated stage I NSCLC reported a median survival of 28 months, and a multi-center study on fractionated radiotherapy reported overall survival medians of 37.6 and 24.1 months for stage IA and IB NSCLC, respectively [18,25]. In comparison, single-center studies on stage I NSCLC patients reported overall survival medians of 33, 33.8, and 68 months after RFA, microwave ablation, and cryoablation respectively [24,26,27].

The primary advantage of lung ablation over traditional alternatives is sparing patients of radiation exposure. Radiation-induced heart disease is a well-described adverse effect with reported incidence rates as high as 33% among radiotherapy patients [28]. A study of 803 SBRT-treated early-stage NSCLC patients found that radiation doses to the left atrium and superior vena cava were significantly associated with non-cancer death [29]. This is especially concerning for patients with stage I NSCLC, as they typically live long enough for non-cancer cardiac death to be a major risk. Such toxicity over time may partially explain the higher 5-year survival rate of 68% for cryoablation compared to 42%–55% for SBRT (Table 1). A retrospective comparison between RFA and SBRT for single lung tumors found comparable outcomes for 3-year local tumor progression, overall survival rate, and complication rates [30]. However, a retrospective comparison between various thermal ablation techniques and SBRT for stage I NSCLC using data from the National Cancer Database (NCDB) found no significant difference in overall survival rate but a higher rate of adverse effects in thermal ablation patients [31]. Another study using the NCDB reported a slightly lower overall survival rate for stage I NSCLC treated with various thermal ablation techniques compared to SBRT (33.5 vs. 37.7 months) [32]. Because of how ablations are coded in the NCDB, the studies could not determine outcomes for each of the techniques individually, which may vary significantly (Table 1). There have been no studies comparing specifically cryoablation to SBRT or fractionated radiotherapy. Importantly, there have also been no clinical trials to definitively compare ablative techniques to each other or to radiation therapy.

### 3.2. Metastases to the Lungs

**Figure 3:**
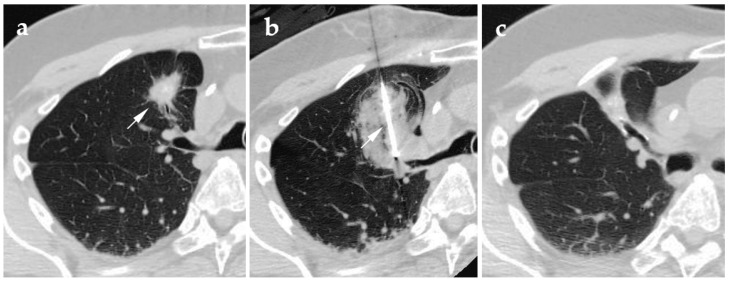
58-year-old male presenting with colorectal metastases to the lung, biopsy proven post-radiation therapy recurrence. (**a**) Supine axial pre-ablation CT with the white arrow indicating the lesion to be ablated; (**b**) supine axial CT of the probe (white arrow) placement during the tumor cryoablation; (**c**) supine axial follow-up CT at 2 years post-ablation.

**Figure 4:**
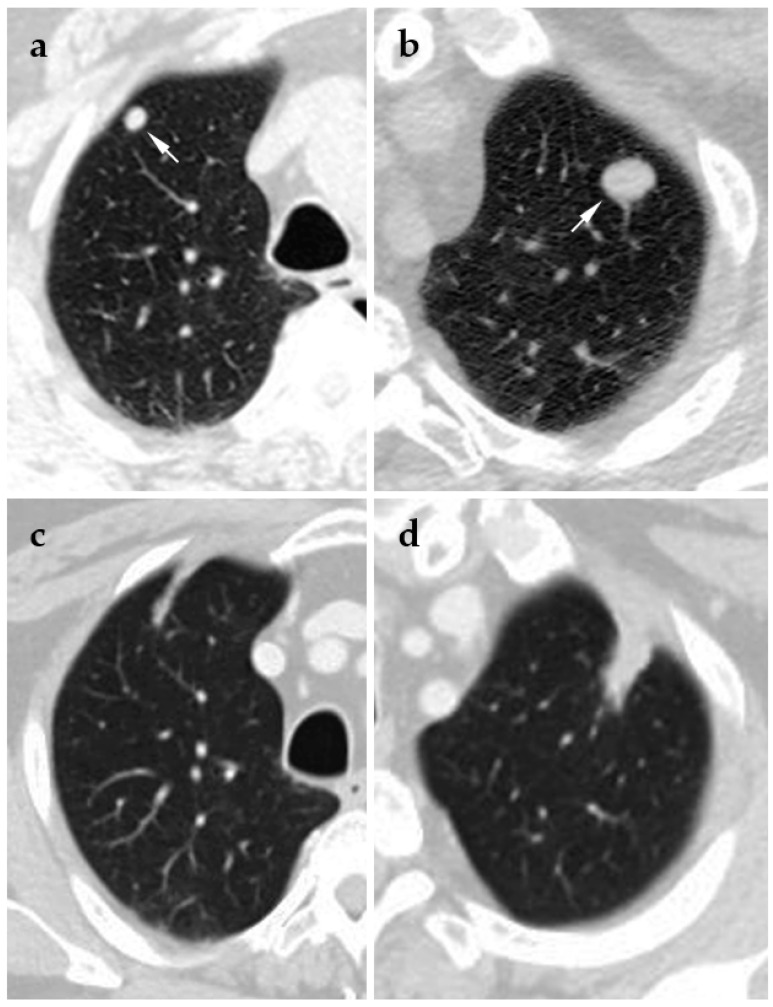
65-year-old male presents with a history of end-stage liver disease secondary to chronic hepatitis C complicated by hepatocellular carcinoma. Patient underwent an orthotopic liver transplantation. Four years after the transplant, the patient underwent a biopsy of a lung lesion, which was positive for metastatic hepatocellular carcinoma. The patient then underwent cryoablation of the left lung, followed by cryoablation of the right lung one month following initial ablation. When treating bilateral lung lesions, the authors ablate one lung at a time to avoid the risk of bilateral pneumothorax. (**a**) Supine axial pre-ablation CT of the right upper lobe nodule (white arrow indicates the lesion to be ablated); (**b**) supine axial pre-ablation CT of the left upper lobe nodule (white arrow indicates the lesion to be ablated); (**c**) supine axial follow-up CT of the right upper lobe at 18 months post-ablation; (**d**) supine axial follow-up CT of the left upper lobe at 18 months post-ablation.

The lungs are the second most frequent site of metastases, which commonly result from lymphatic and hematogenous spread of melanoma as well as primary breast, colorectal, renal, head and neck, and uterine carcinomas, among others [33]. Local treatment of lung metastases is widely practiced, as large retrospective analyses consistently demonstrate the survival benefit of metastasectomy (Figure 3 and Figure 4) [3,34,35]. The Society of Thoracic Surgeons endorses surgical metastasectomy for appropriately selected patients and recognizes SBRT and thermal ablation as reasonable alternatives, especially for patients with surgical contraindications [36]. Curative ablation of metastases is generally indicated for patients with no more than three unilateral lung lesions and five total lung lesions with a maximum tumor diameter of 3 cm for multiple lesions and 5 cm for a single lesion [37].

Table 2 compares the survival rates of various treatment modalities for lung meta-states. A prospective study on 1037 RFA-treated lung metastases in 566 patients found that patients could be safely treated up to four times and reported a median overall survival of 62 months, a 5-year overall survival rate of 51.5%, and a 4-year local efficacy of 89% [38]. Prognoses varied based on lesion size, number of metastases, disease-free interval, and primary cancer, in which colon cancer and sarcoma had the highest (56.0%) and lowest (41.5%) 5-year overall survival rates, respectively. In comparison, a systematic review reported 5-year overall survival rates from 27% to 68% for colorectal cancer treated with lung metastasectomy [39]. The 1-year interim analysis of the ECLIPSE prognostic study on cryoablation-treated lung metastases reported a local efficacy rate of 94.2% at 12 months, with 18.8% of cases leading to pneumothorax requiring chest tube placement. In comparison, the 1-year local efficacy reported in the RFA-treated lung metastases study was 94.1%, with 38.9% of all cases leading to pneumothorax requiring chest tube placement. The SOLSTICE phase II trial for cryoablation-treated lung metastases reported a 2-year local efficacy rate and overall survival rate of 77.2% and 86.6%, respectively. The rate of pneumothorax requiring chest tube placement was 26% [40]. In comparison, the RFA study reported a 2-year local efficacy rate and overall survival rate of 84.5% and 79.4%, respectively. The evidence thus far suggests that cryoablation may be just as effective and safe as RFA for lung metastases.

Thermal ablation offers several advantages over surgery and SBRT in treating lung metastases. Because there is no difference in outcome for this indication between wedge resection, lobar resection and pneumonectomy, the ideal treatment would destroy the tumor with minimal parenchymal loss [40,41,42]. Image-guided ablation delivers precisely-placed tumor destruction without being limited by the segmental anatomy of the lungs, thereby enabling providers to spare tissue peripheral to the lesion [43,44,45]. Given the frequency of recurrence after local therapy for metastatic disease, the repeatability of lung ablation is a significant asset [38]. In contrast, the accumulation of radiation with SBRT is of great concern [29]. A systematic review of colorectal cancer metastases to the lungs limited by variable reporting methods and lack of clinical trials reported overall survival medians and 5-year survival rates of 33–51 months and 34.9–45% for RFA compared to 27–72 months and 29–71.2% for metastasectomy [46]. Reported iatrogenic mortality and morbidity rates were 0% and 22–45% for RFA compared to 0–2.4% and 0–14.5% for metastasectomy. Thus, low risk of death is another advantage. No such comparisons with SBRT have been reported.

### 3.3. Palliative

**Figure 5:**
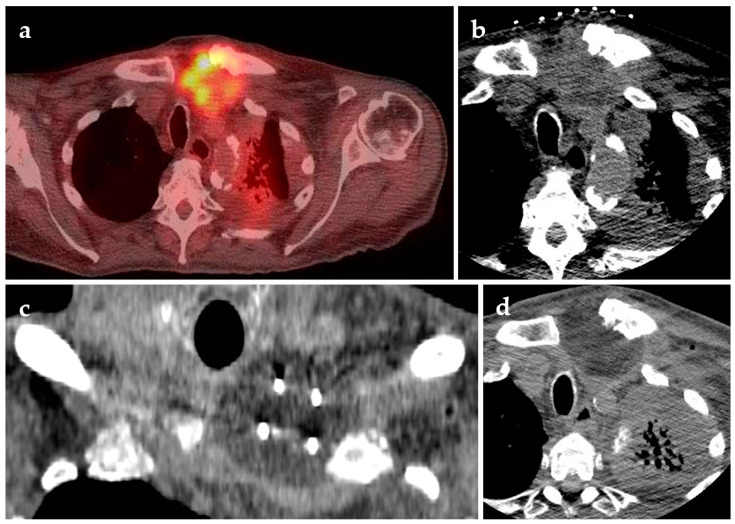
78-year-old female diagnosed with squamous cell carcinoma appearing as a left supraclavicular mass was treated with palliative ablation. The patient presented with left upper extremity pain, weakness and hoarseness of voice, as well as post radiation changes in the skin. (**a**) Supine axial FDG-PET scan demonstrating FGD-avid metastasis; (**b**) supine axial CT used for treatment planning; (**c**) supine coronal CT shows the probe positioning for cryoablation; (**d**) supine axial follow-up CT post-ablation.

Lung tumors can cause respiratory symptoms and severe pain. A wide variety of palliative treatments, including opioids, is available for the ultimate goal of improving quality of life [47]. Patients who, according to the aforementioned guidelines, do not qualify for curative ablation can still benefit from the palliative effects of lessening tumor burden (Figure 5). As such, the indications for palliative ablation of lung lesions are broad and patient selection is often at the discretion of the provider [48,49,50]. As lesions over curative size limits are more difficult to completely ablate, larger tumors require more intense treatment in the form of multiple applicators, multiple ablative sessions, or combination with radiotherapy or surgery [37,51].

In a study on RFA-treated stage I-IV NSCLC, the 20 palliative-intent patients had response rates of 80% (4 of 5) for hemoptysis, 36% (5 of 14) for chest pain, 36% (4 of 11) for dyspnea and 25% (2 of 8) for cough. Their overall survival mean was 5.6 months, compared to 21.2 months for the curative group [51]. Ablation in palliative settings may nevertheless prolong life. In a study on RFA-treated unresectable renal cell carcinoma metastases to the lungs, the 24 palliative patients had a 5-year overall survival rate of 52%, compared to 100% in the curative group [49]. Considering that the reported overall survival rates for lung metastasectomy of renal cell carcinoma range from 35.5% to 54%, the palliative ablation results are promising [52,53]. Furthermore, a study on stage IV NSCLC found the 31 cryoablation patients to have a median overall survival of 14 months compared to 7 months in the group treated with other palliative therapies (radiation and chemotherapy) [54].

### 3.4. Pleural Lesions

Although the focus of this review is on ablation of pulmonary lesions, the intimate relationship between lung and pleura warrants discussion on the recent developments in image-guided percutaneous pleural ablation. Due to its comparatively limited discomfort and moderate sedation necessity, cryoablation is the preferred ablative therapy for pleural lesions [55].

The most common primary pleural malignancy, malignant pleural mesothelioma is typically treated with a tri-modality therapy consisting of surgery, adjuvant chemotherapy, and radiation [56]. However, recurrence rate is high at 4–41% locally and 27–84% overall, contributing to the low overall survival median after tri-modality therapy of 14–33 months [57,58,59,60]. Traditionally, patients with recurrence are treated with second-line chemotherapy, additional radiation, or palliative care. Abtin et al. reported on treatment of 24 patients with 110 pleural cryoablations for recurrent mesothelioma tumors, for which the 3-year local control rate was 73.7% [61].

Thymoma is typically surgically resected, but 10–30% of cases recur with 90% appearing in the pleura [62]. Further resection of pleural recurrence is challenging and dangerous, while alternative treatments such as chemotherapy and radiation have significant adverse effects [63,64]. Abtin et al. published a case series of 25 thymoma recurrence lesions in five patients treated with cryoablation, in which 90% (18 of 20) of cases were recurrence-free upon follow-up (median 331 days) [65]. These recent publications demonstrate the feasibility of adding cryoablation to the highly limited selection of therapies available for pleural malignancies.

## 4. Techniques

### 4.1. Cryoablation

The Joule-Thomson effect describes how, at room temperature, all gases except hydrogen, helium, and neon cool when moving from an area of high pressure to an area of low pressure through an orifice [66]. Exploiting this phenomenon, cryoablation systems freeze tissue by delivering pressurized argon gas to the tip of a cryoprobe, where it is passed through an orifice before entering a low-pressure section in a closed circuit. Helium exhibits the opposite effect at room temperature and is used by cryoablation systems to thaw tissue [67]. Approaching a target temperature of −40 °C, cryoablation initially forms ice crystals in the extracellular space, which increases extracellular tonicity and causes osmotic damage to surrounding cells [68,69]. Intracellular ice eventually forms, which ruptures both the plasma and organelle membranes [70]. Indirect cell death occurs afterward, in which damaged blood vessels thrombose, causing ischemia as well as inflammation [71].

**Figure 6:**
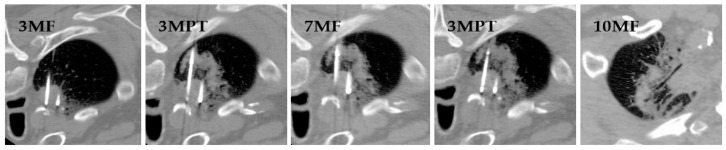
CT-visualization of a modified triple-freeze cryoablation protocol: 3 min freezing (3MF), 3 min passive thawing (3MPT), 7 min freezing (7MF), 3 min passive thawing (3MPT), and 10 min freezing (10MF). This is followed by active thawing and probe removal. Note that the patient was flipped from prone to supine position after 3 min freezing in the last image, as this is likely to decrease the risk of pneumothorax.

Cryoablation is usually performed under moderate sedation for peripheral lesions and general anesthesia for cases involving central lesions or high risk of bleeding. While the treated lung is under continuous positive airway pressure, the other lung is mechanically ventilated [72]. The number of cryoprobes to be inserted depends on lesion size, with probes placed no more than 2 cm from each other and no more than 1 cm from the tumor margin [73]. Using CT-guidance, the cryoprobes are inserted percutaneously until the tip is placed at or alongside the target lesion. As a lesion’s firmness may prevent direct penetration, small lesions are bracketed with several cryoprobes rather than penetrated. Different freeze–thaw cycle schemes have been developed in attempts to maximize tumor destruction and safety. The authors prefer the modified triple-freeze protocol, which is 3 min of freezing, 3 min of passive (without helium) thawing, 7 min of freezing, 3 min of passive thawing, and 10 min of freezing (Figure 6) [74]. The triple-freeze protocol, due to lower continuous thaw time, has been shown to decrease hemorrhage and allow for earlier ice ball detection compared to the older double-freeze protocol [75,76]. The modified triple-freeze protocol further reduces thaw time to lower hemorrhage risk. Although the area of lung necrosis is difficult to predict based on CT-visualization, ice balls are monitored to ensure they do not encroach on vulnerable structures. After probe removal, the patient is screened for complications by chest CT. Patients are typically then discharged home on the same day unless complications warrant admission.

Advantages of cryoablation over other thermal ablation techniques include the preservation of the collagenous matrix of the lungs and other tissues, allowing for its safe usage near the airways, pericardium, blood vessels, and bone [55,77]. Uniquely, cryoablation is also safe to use in the pleura, while RFA and microwave ablation are known to cause bronchopleural fistulas [78,79]. Cryoablation is also associated with less post-procedural pain compared to RFA and microwave ablation [80,81]. Ice ball visualization, though difficult to correlate with ablative zone on lung CT, has some monitoring utility [82]. Limitations include longer setup times than RFA and microwave ablation and longer procedure times, which result in increased CT radiation exposure.

### 4.2. Radiofrequency Ablation

RFA delivers radiofrequency waves in the 375 to 500 kHz range to an area surrounding an electrode, which is inserted in a similar manner as a cryoprobe. A radiofrequency generator coupled to the electrode produces a voltage between the electrode (acting as the cathode) and grounding pads (acting as the anode) placed on the patient’s thighs. As an alternating current is generated, electric field lines are established with the highest energy flux around the electrode tip due to its small cross-sectional area compared to the pads [83]. The oscillating electric field generates frictional energy by colliding electrons with surrounding molecules, generating heat sufficient for cell death (>60 °C) [84].

The main advantage of RFA is its well-documented efficacy and safety, as it is the most widely used ablation technique for solid tumors and holds Food and Drug Administration (FDA) approval for treating lung cancer [37,55]. However, tissue charring may impede ablation. RFA relies on an electrically conductive path between the electrode and grounding pads and thus may not function in areas of high electrical resistance [85]. Ventilation and perfusion in areas with large airways or vessels may remove heat and limit ablation, which is not an issue for cryoablation. There is also evidence of interference with cardiac electrophysiology and pacemaker devices [86].

### 4.3. Microwave Ablation

Unlike RFA, which operates through a current traveling from a cathode to an anode placed on different parts of the body, microwave ablation directly applies an electromagnetic field at 900–2500 MHz to surrounding tissue via a dielectric antenna placed in a similar manner as a cryoprobe [87]. Water and other polar molecules continuously realign with the electrical oscillations to produce kinetic energy and ablative heat.

Microwave ablation is more versatile than RFA because it does not depend on an electrically conductive path. Furthermore, microwaves are not limited by tissue thermal conductivity or charred tissue [51,88]. Capable of inducing higher temperatures at a faster rate than RFA, it can better treat highly ventilated or perfused areas. However, the wide variety of different device designs complicates training and reporting [89].

### 4.4. Laser Ablation

Laser ablation delivers ablative heat to tissues by positioning laser catheters around the lesion of interest. The typical protocol involves introducing a 21-gauge Chiba needle to the lesion, and using a neodymium-doped yttrium aluminum garnet (Nd:YAG) laser to deliver 1064 nm light via a fiberoptic cable [90]. Advantages of this technique include the use of thin-caliber applicators and lower costs than other ablative methods [91]. The relatively small areas of necrosis induced by laser ablation may allow for better control and predictability [92]. Like RFA, tissue charring is a limitation to this technique. Laser ablation of lung tumors is not widely practiced and literature on its effectiveness and safety is extremely scarce.

### 4.5. Irreversible Electroporation

Irreversible electroporation (IRE), the only non-thermal ablation technology, uses direct, microseconds to milliseconds-long electric pulses to generate brief electric fields that irreversibly rupture cell membranes and cause necrosis [93]. Pulses are delivered via electrodes or probes that are placed in a similar manner as cryoprobes. The mechanism by which the electric fields rupture membranes is not completely understood as this process often varies with application [94,95]. Notably, IRE differs from thermal ablation technologies in that it establishes a specific demarcation area that only affects the cell membrane and no other tissues of interest [94,95]. Theoretical advantages of IRE include overcoming the heat sink of highly ventilated or perfused areas and safety in treating lesions close to blood vessels [94,95]. However, investigation of this application has largely been abandoned after a phase II trial of lung malignancies treated with irreversible electroporation was stopped prematurely due to a high recurrence rate of 61% within 1 year of treatment [96].

## 5. Contraindications and Complications

Image-guided percutaneous lung ablation is well-tolerated and has relatively few contraindications. Thermal ablation should be avoided in patients with poorly controlled infection, severe pulmonary fibrosis, severe hemorrhage tendency, severe organ dysfunction, severe anemia, severe metabolic disorders, extensive extrapulmonary metastases with an expected survival of less than 3 months, or an Eastern Cooperative Oncology Group (ECOG) score greater than 3. Additionally, patients with pacemakers should not receive RFA [37].

According to a large retrospective study of 3344 lung tumor patients treated with thermal ablation using data from the Nationwide Inpatient Sample database, the most common complications are pneumothorax (38.4%), pneumonia (5.7%), effusion (4%), in-hospital mortality (1.3%), and conversion to thoracoscopy or thoracotomy (0.9%) [97]. The study found that the risk of pneumothorax was not associated with increased mortality. To decrease the risk of pneumothorax, the authors immediately flip the patient to the opposite side after probe removal (Figure 6). Even when a chest tube is necessary for pneumothorax, patients are typically discharged the day after ablation [43]. Although the possibility of pneumothorax appears to be a disadvantage compared to SBRT, radiation therapy often requires biopsies or placement of fiducial markers, both of which carry risk for pneumothorax. Moreover, ablation can be performed in conjunction with core-needle biopsy through the same entry site, thereby yielding more patient benefit for virtually the same amount of risk [98].

Another documented complication is hemorrhage, with reported incidences following RFA ablation ranging from 6% to 18% [99,100]. The vast majority of ablation-induced hemorrhages are self-limiting, although there have been reports of severe and fatal incidents [101]. Practitioners should be mindful of avoiding intercostal artery injury during probe placement by choosing to place probes away from the inferior margin of the rib. In the case of arterial injury during initial probe placement, the authors highly recommend removing the probe before penetrating the parietal pleura.

Systemic air embolism is a rare complication of lung ablation, mostly reported as single cases [102,103,104]. In a review of complications, Okuma et al. reported one case of air embolism out of 112 RFA procedures in 57 patients [105,106]. None of the reported incidents were fatal. In the event of a systemic air embolism, 100% oxygen should be administered immediately in order to oxygenize and promote resorption of the air [101]. Contraindications and complications are summarized in Table 3.

## 6. Conclusions

Percutaneous image-guided lung ablation presents a promising treatment option for patients with a variety of lung tumors. Investigating potential applications to new indications is an exciting and active area of research. As impressive outcomes for cryoablation emerge, there is a need for randomized controlled trials comparing 5-year survival between lung tumor patients treated with SBRT and cryotherapy. The management of complex patients should be undertaken by a multidisciplinary team in order to optimize early detection and incorporate effective therapeutic options. Doing so would likely result in an increased need for less invasive yet efficacious modalities, such as thermal ablation.

## Figures and Tables

**Table 2:** Comparison of survival rates between surgical metastasectomy, radiofrequency ablation, and cryoablation for metastases to the lungs.

Modality	Description	2-Year Survival	5-Year Survival	Median Survival	Sample Age (Mean, Median, Range)
Surgical metastasectomy [39]	The surgical removal of visible and palpable cancerous tissue	NA	27–68%	18.5–72 months	55–65, NA, NA
Radiofrequency ablation [38]	A probe delivers high-energy radio waves to the tumor, heating the tumor and destroying cancerous cells	79.4%	51.5%	62 months	62.7, NA, 17–92
Cryoablation [40]	A cryoprobe delivers gas to the tumor, freezing the tumor and killing any cancerous cells	77.2%	NA	NA	NA, 65, 12–85

NA = Not Available.

**Table 3:** Contraindications to and complications of lung ablation.

Contraindications	Complications
Poorly controlled infection	Pneumothorax
Severe pulmonary fibrosis	Pneumonia
Severe hemorrhage tendency	Effusion
Severe organ dysfunction	In-hospital mortality
Severe anemia	Conversion to thoracoscopy/thoracotomy
Severe metabolic disorders	Hemorrhage
Extensive extrapulmonary metastases with an expected survival of less than 3 months	Systemic air embolism
Eastern Cooperative Oncology Group (ECOG) score greater than 3	
Pacemaker (radiofrequency ablation)	
